# Clonal CTX-M-15-Producing *Escherichia coli* ST-949 Are Present in German Surface Water

**DOI:** 10.3389/fmicb.2021.617349

**Published:** 2021-04-12

**Authors:** Linda Falgenhauer, Anja zur Nieden, Susanne Harpel, Jane Falgenhauer, Eugen Domann

**Affiliations:** ^1^Institute of Hygiene and Environmental Medicine, Justus Liebig University Giessen, Giessen, Germany; ^2^German Center for Infection Research (DZIF), Partner Site Giessen-Marburg-Langen, Justus Liebig University Giessen, Giessen, Germany; ^3^Institute of Medical Microbiology, Justus Liebig University Giessen, Giessen, Germany

**Keywords:** CTX-M-15, ST-949, ESBL-*E. coli*, water samples, WGS

## Abstract

Extended-spectrum beta-lactamase (ESBL)-producing bacterial isolates are emerging within the last years. To understand this emergence, a thorough genome-based analysis of ESBL isolates from different sources (One Health approach) is needed. Among these, analysis of surface water is underrepresented. Therefore, we performed a genome-based analysis of ESBL-producing *Escherichia coli* isolates from surface water samples. Water samples were collected from eleven different surface water sites (lakes, river). ESBL-producing *E. coli* were recovered from these samples using filters and chromogenic media. Whole-genome sequencing of ESBL-producing *E. coli* was performed followed by determination of the multilocus sequence type (ST), ESBL-type, and virulence genes. Phylogenetic analysis was done using single nucleotide analysis. From all water samples taken, nineteen ESBL-producing *E. coli* were recovered. All of them harbored an ESBL gene. Nine different multilocus STs were determined, among which ST-949 was the ST detected most frequently. Phylogenetic analysis of ST-949 isolates revealed that all those isolates were closely related. In addition, they harbored an identical chromosomal insertion of *bla*_*CTX–M–15*_, indicating a clonal relationship among these isolates. Genetic comparison with isolates from all over the world revealed that these isolates were closely related to human clinical isolates derived from New Zealand and Sweden. An ESBL-producing *E. coli* ST-949 clone was detected in German surface waters. Its close relationship to human clinical isolates suggests its ability to colonize or even infect humans. Our findings reveal that water sources indeed may play a hitherto underreported role in spread of ESBL-producing isolates.

## Introduction

Extended-spectrum beta-lactamase (ESBL)-producing bacterial isolates are emerging in the last years (Peirano and Pitout, 2019). The spread of ESBL-producers is a clear One Health issue, as they have been found to be present in different sources, animals, humans, and environment (Hooban et al., 2020). This is true for Germany as well. In Germany, 6.3% of humans are colonized with ESBL-producing *Escherichia coli* isolates (Valenza et al., 2014). In diseased food-producing animals, the prevalence of ESBL-producing *E. coli* ranges between 0.8 and 11.2% depending on the animal species (Michael et al., 2017).

The commonly accepted opinion is that all different sources play a role in the spread of ESBL-producing bacteria. To be able to track the transfer routes of ESBL-producers among different sources, a thorough understanding of the epidemiology of these bacteria is needed. The method of choice to perform an in-depth epidemiological analysis is to use whole genome sequence-based methods. They have been used a lot in human and veterinary medicine (in particular to track outbreaks), but genome-based data from water sources are still very rare. Few epidemiological studies have been performed to analyze the genomes of ESBL-producing isolates from water samples. These studies showed a high identity between ESBL producers from water samples and clinical samples indicating a spread from either clinical to water sources or vice versa (Fagerström et al., 2019).

In order to gain more insight into this topic, an investigation was performed that included water samples from official and unofficial bathing sites at lakes and a river in Hesse, Germany.

## Materials and Methods

### Sampling Procedure

During the bathing season 2018, samples were taken from swimming lakes in Hesse (*n* = 10). According to the European Bathing Water Directive (BWD; EG 2006/7) the sites were checked at least monthly for the presence of coliform bacteria. Additionally, samples from unofficial bathing sites of the Hessian river Lahn around Marburg and Giessen were taken (*n* = 9). Procedures for sampling as well as preparation, filtration, and enumeration were performed conforming with the DIN EN ISO 9308-2 (K6-1) 07-2014, DIN EN ISO 19458 (K19), DIN EN ISO 8199 (K20) 01-2008, and DIN EN ISO 9380-1: 2014 (K12) regulations within 24 h.

### Characterization of ESBL-Producing Isolates

For detection of ESBL-producing isolates, water samples were filtered and the filters put onto Brilliance^TM^ ESBL chromogenic medium (OXOID, Wesel, Germany). For isolates growing on the chromogenic agar, species confirmation was performed using MALDI-TOF-MS (Biomérieux, Nürtingen, Germany). Antibiotic susceptibility testing and ESBL phenotype confirmation was performed using the VITEK 2 System (AST-N263 cards, Biomérieux, Nürtingen, Germany). Classification of the antibiotic resistance/susceptibility was performed according to EUCAST criteria^1^.

### Whole-Genome Sequencing

Short-read whole genome sequencing was performed for all *E. coli* isolates growing on the chromogenic medium (*n* = 21). DNA from overnight cultures was isolated using the Purelink genomic DNA kit (ThermoFisher, Dreieich, Germany). Short read sequencing was performed on a NextSeq 500 machine (Illumina, Eindhoven, Netherlands) using a Nextera XT sequencing library with an average read length of 115 nt and an average coverage of 33.5 x. Raw reads were processed using the ASA^3^P pipeline using default parameters (Schwengers et al., 2020).

Long-read sequencing of a representative *E. coli* ST-949 isolate (EDCC5518) was performed using the Nanopore technology. The library was prepared using the native barcoding kit (EXP-NBD103, Oxford Nanopore Technologies Ltd., Oxford, United Kingdom) and 1D chemistry (SQK-LSK108). Sequencing was performed using the SpotON Flow Cell Mk I R9 Version (FLO-MIN106) on a MinION/MinIT machine with an average read length of 4,137 nt. Basecalling was performed directly on the MinIT machine. Demultiplexing was performed using Porechop (v. 0.2.3^2^). Hybrid assembly was performed using Unicycler (v. 0.4.7) (Wick et al., 2017) and the short and long reads with default parameters.

### Genome-Based Analyses

*In silico* multilocus sequence typing of *E. coli* isolates was performed using the scheme presented by Wirth et al. (2006). Antibiotic resistance genes, plasmid incompatibility groups and *fimH* types were determined using the bacterial analysis pipeline of the Center for Genomic Epidemiology^3^. Insertion elements were determined using ISFinder (Siguier et al., 2006). Virulence gene determination was performed using ASA^3^P (Schwengers et al., 2020). Comparative genome analysis was performed using the HarvestSuite package (Treangen et al., 2014). Publicly available assembled *E. coli* genomes of the multilocus sequence type (ST) ST-949 were downloaded using Enterobase (as of 8th June 2020, Supplementary Table 1) (Zhou et al., 2020). Geographical representation of sampling sites was visualized using MicroReact (Argimón et al., 2016).

## Results and Discussion

### Detection and Phenotypic Characterization of ESBL-Producing *Escherichia coli* Samples

**FIGURE 1:**
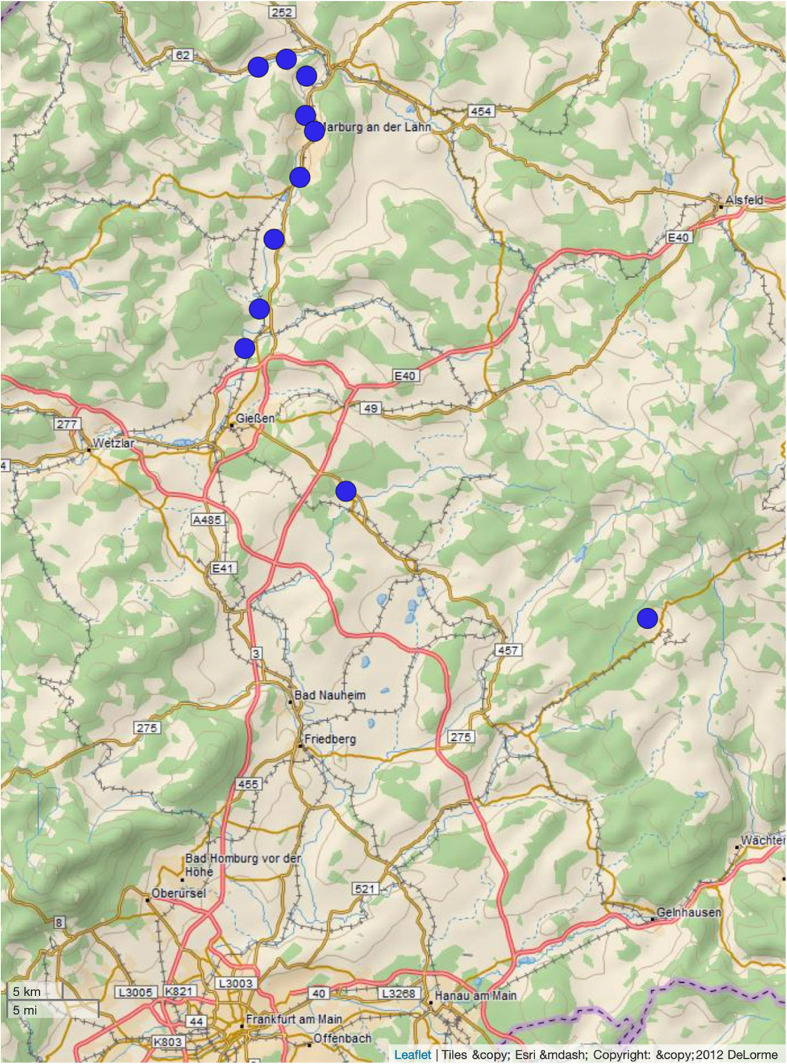
Distribution of sampling sites. The figure was generated using microreact (Argimón et al., 2016).

During the bathing season of 2018 (June–August), fifty-five samples from nineteen sampling sites were collected. Of these samples, forty-four did not show growth of isolates on ESBL chromogenic agar. Notably, the 2018 summer was a comparatively hot summer^4^ resulting in low water levels. From the remaining water samples (*n* = 11, Figure 1), nineteen ESBL-producing bacterial isolates were detected (Table 1). Only *E. coli* isolates were detected. Environmental data and characterization of the sampling sites are shown in Table 1.

**TABLE 1:** Environmental data and enumeration results of sampling sites with ESBL-positive samples.

**Sampling site #**	**Isolate #**	**Species**	**Site**	E. coli* [CFU/100 ml]	Enterobacter* [CFU/100 ml]	**Temperature air [°C]**	**Temperature water [°C]**	**Sampling date**	**Sampling time**
1	EDCC5518	*Escherichia coli*	Bathing lake	77	15	21	20	19.06.18	09:20
	EDCC5519	*Escherichia coli*							
	EDCC5520	*Escherichia coli*							
	EDCC5522	*Escherichia coli*		15	46	23	24	17.07.18	09:00
2	EDCC5521	*Escherichia coli*	Bathing lake	<15	15	23	24	16.07.18	09:30
6	EDCC5523	*Escherichia coli*	Bathing lake	109	161	28	27	07.08.18	10:00
	EDCC5524	*Escherichia coli*							
10	EDCC5525	*Escherichia coli*	River	<15	<15	27	21	08.08.18	9:41
11	EDCC5526	*Escherichia coli*	River	30	<15	28	22	08.08.18	10:21
13	EDCC5527	*Escherichia coli*	River	1,509	144	27	19	08.08.18	11:38
14	EDCC5528	*Escherichia coli*	River	1,749	94	25	19	08.08.18	12:09
	EDCC5529	*Escherichia coli*							
	EDCC5530	*Escherichia coli*							
15	EDCC5531	*Escherichia coli*	River	5,352	640	28	23	08.08.18	12:31
16	EDCC5532	*Escherichia coli*	River	1,931	197	32	23	08.08.18	12:58
	EDCC5533	*Escherichia coli*		1,931	197	32	23	08.08.18	12:58
17	EDCC5534	*Escherichia coli*	River	110	<15	32	24	08.08.18	13:36
	EDCC5535	*Escherichia coli*		110	<15	32	24	08.08.18	13:36
19	EDCC5536	*Escherichia coli*	River	77	<15	32	24	08.08.18	15:53
	EDCC5537	*Escherichia coli*							
	EDCC5538	*Escherichia coli*							

*Enumeration performed based on DIN EN ISO 9308-2 (K6-1) 07-2014 regulation; EDCC, ED culture collection.

For all *E. coli* isolates growing on the chromogenic plates, the ESBL phenotype was confirmed. Phenotypic resistance to antibiotics other than beta-lactams was detected very seldom and included resistance to fluoroquinolones (4/21) and trimethoprim/sulfamethoxazole (5/21) (Supplementary Table 2). The isolates were not resistant to carbapenems. According to the classification proposed by Magiorakos et al. (2012), all isolates were multidrug-resistant (resistant to ≥3 different antibiotic classes; Supplementary Table 2).

### Genome-Based Analysis of *Escherichia coli* Isolates

All *E. coli* isolates harbored an ESBL gene (Table 2). The most common ESBL gene detected was *bla*_*CTX–M–15*_ (*n* = 17) followed by *bla*_*CTX–M–1*_ (*n* = 2) and *bla*_*CTX–M–27*_ (*n* = 2). The predominance of *bla*_*CTX–M–15*_ in our study is concordant with the results in other studies performed in Europe (Kittinger et al., 2016; Jorgensen et al., 2017). The *E. coli* isolates encoded other antibiotic resistance genes conferring resistance to aminoglycosides (7/21), fluoroquinolones (15/21, *qnrS1*), sulfonamide (5/21), trimethoprim (5/21), and tetracycline (2/21) (Table 2). In concordance with previous reports (Rodríguez-Martínez et al., 2011), the presence of *qnrS1* did not lead to high-level fluoroquinolone resistance (MIC > 0.5 mg/L) in our isolates.

**TABLE 2:** Results of the genome-based analysis of the ESBL-producing *E. coli* isolates.

**Isolate**	**ST**	**Aminogly coside**	**Beta- lactam**	**Macrolide**	**Phenicol**	**Quinolone**	**Sulfonamide**	**Tetracycline**	**Trime thoprim**	***fimH* type**	**Plasmid incom patibility groups**
EDCC5518	949		*bla*_*CTX–M–15*_			*qnrS1*				H121	IncI1 and p0111
EDCC5519	949		*bla*_*CTX–M–15*_			*qnrS1*				H121	IncI1
EDCC5520	949		*bla*_*CTX–M–15*_			*qnrS1*				H121	IncI1 and p0111
EDCC5521	3,246		*bla*_*CTX–M–1*_							H65	IncFIA, IncI1, IncFIB (AP00 1918), IncFII(29), and ColRNAI
EDCC5522	949		*bla*_*CTX–M–15*_			*qnrS1*				H121	IncI1 and IncA/C2
EDCC5523	949		*bla*_*CTX–M–15*_			*qnrS1*				H121	IncI1
EDCC5524	949		*bla*_*CTX–M–15*_			*qnrS1*				H121	IncI1
EDCC5525	949		*bla*_*CTX–M–15*_			*qnrS1*				H121	IncI1
EDCC5526	949		*bla*_*CTX–M–15*_			*qnrS1*				H121	IncI1
EDCC5527	1,490	*aadA1*	*bla*_*CTX–M–15*_			*qnrS1*			*dfrA1*	H25	IncFII, IncFIB (AP00 1918), IncFII (pCoo), and IncB/O/K/Z
EDCC5528	2,797		*bla*_*CTX–M–15*_			*qnrS1*				H54	IncFII (pHN7A8) and IncB/O/K/Z
EDCC5529	131	*strA, strB*	*bla*_*CTX–M–15*_, *bla*_*TEM–1B*_	*mph(A)*			*sul2*	*tet(A)*		H41	IncFII(29), IncFIB (AP00 1918), and Col156
EDCC5530	155	*aadA5*	*bla*_*CTX–M–1*_				*sul2*		*dfrA17*	N.D.	IncI1 and IncFII(pCoo)
EDCC5531	226	*aadA1*	*bla*_*CTX–M–15*_, *bla*_*OXA–1*_		*catA1*			*tet(B)*		H41	IncFII, ColRNAI, and Col(MG828)
EDCC5532	949		*bla*_*CTX–M–15*_			*qnrS1*				H121	IncI1
EDCC5533	949		*bla*_*CTX–M–15*_			*qnrS1*				H121	IncI1, Col8282, ColRNAI, Col156, and Col(MG828)
EDCC5534	949		*bla*_*CTX–M–15*_			*qnrS1*				H121	IncI1
EDCC5535	131		*bla*_*CTX–M–27*_							H30	IncFII (pRSB107), IncFIA, IncFIB(AP 001918), Col8282, Col156, and Col(MG828)
EDCC5536	295	*aadA5*	*bla*_*CTX–M–27*_, *bla*_*TEM–1B*_	*mph(A)*			*sul1*		*dfrA17*	H54	IncFII (pRSB107), IncFIB (AP00 1918), IncFII (pCoo), IncY, and ColRNAI
EDCC5537	1,431	*aadA1, aadA2, strA, strB*	*bla*_*CTX–M–15*_, *bla*_*TEM–1B*_		*cmlA1*	*qnrS1*	*sul2, sul3*		*dfrA12*	H32	IncI1, IncX1, IncY, and Col156
EDCC5538	1,431	*aadA1,aadA2, strA, strB*	*bla*_*CTX–M–15*_, *bla*_*TEM–1B*_		*cmlA1*	*qnrS1*	*sul2, sul3*		*dfrA12*	H32	IncI1, IncX1, IncY, and Col156

*EDCC, ED culture collection; N.D., not detected.*

**FIGURE 2:**
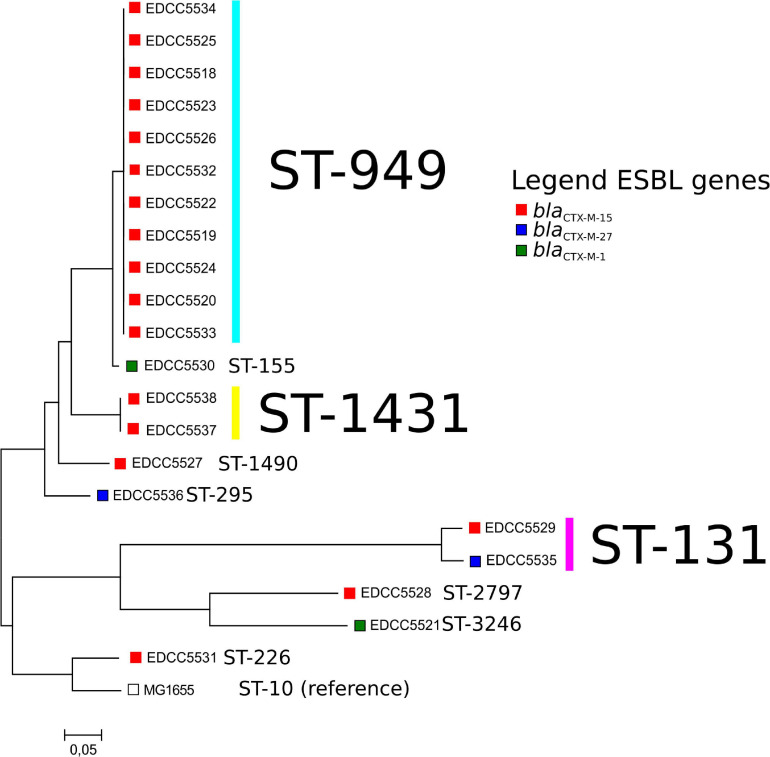
Core-genome-based comparison of the *E. coli* isolates analyzed in this study. Boxes depict the ESBL gene present in these isolates. For a representation of the isolate geographical location and the phylogeny, please see the following link: https://microreact.org/project/bELapwXL1Fi8ZdznzZHF1H.

Multilocus sequence typing revealed that nine different STs were present (Table 2 and Figure 2). Of these, three were detected more than once: ST-949 (*n* = 11), ST-131 (*n* = 2), and ST-1431 (*n* = 2). *E. coli* ST-949 and ST-1431 isolates harbored *bla*_*CTX–M–15*_, while *E. coli* ST-131 isolates harbored *bla*_*CTX–M–15*_ or *bla*_*CTX–M–27*_.

To our knowledge, *E. coli* ST-949 have been reported in only five publications worldwide, indicating that this ST is less frequent and might represent an emerging clone (Oh et al., 2014; Potron et al., 2017; Potel et al., 2018; Fagerström et al., 2019; Sedrati et al., 2020). The total number of publicly available *E. coli* ST-949 isolates in the Enterobase database is 41 [as of 11th August 2020 (Zhou et al., 2020)], a very low number compared with frequent multilocus STs as e.g., ST-131 (*n* = 9202, as of 11th August 2020).

ESBL-producing ST-1431 *E. coli* isolates have been detected more often in animal sources (livestock, pets, wild animals) than in humans (Rocha-Gracia et al., 2015; Bachiri et al., 2017; Seiffert et al., 2017).

In this study, we detected two ST131 isolates. *E. coli* ST-131 are frequently associated with human clinical infections (Nicolas-Chanoine et al., 2014), in particular those depicting the *fim*H type H30 and harboring CTX-M-15 or CTX-M-27 (Nicolas-Chanoine et al., 2014; Stoesser et al., 2016). EDCC5529 depicted the fimH41 *fim*H-type and harbored *bla*_*CTX–M–15*_. EDCC5535 depicted a fimH30 *fim*H type and characteristic properties of the ST-131 C1-M27 clade (Matsumura et al., 2016): *bla*_*CTX–M–27*_, the GyrA S83L/D87N and ParC S80I/E84V mutations leading to fluoroquinolone resistance and the M27PP1 phage. Therefore, it is a member of the C1-M27 clade usually associated with human isolates (Matsumura et al., 2016; Ghosh et al., 2017). Thus, EDCC5535 might have originated from human sources.

### Deeper Analysis of *Escherichia coli* ST-949 Isolates

The most common ST within the ESBL *E. coli* was ST-949 (Table 2 and Figure 2). Therefore, we analyzed these isolates in more detail. *E. coli* ST-949 is known to be associated with carbapenem-resistance (Potron et al., 2017; Potel et al., 2018) or ETEC pathotypes (Oh et al., 2014). They have been isolated from environmental samples (water samples) collected in Sweden, where the authors could show that the water isolates were highly related with isolates derived from a hospital that was adjoining the water source (Fagerström et al., 2019).

Because *E. coli* ST-949 are known to be pathogenic (Oh et al., 2014), we analyzed all available *E. coli* ST-949 isolates for the presence of virulence genes. The ST-949 isolates from this study harbored only ExPEC virulence genes (e.g., iron acquisition genes, Enterobactin, Supplementary Figure 2). The isolates detected in New Zealand and Sweden harbored the same sets of virulence genes. Other ST-949 harbored also toxins (Shigatoxin) and hemolysins indicating that ST-949 isolates differ widely in their virulence capabilities.

**FIGURE 3:**
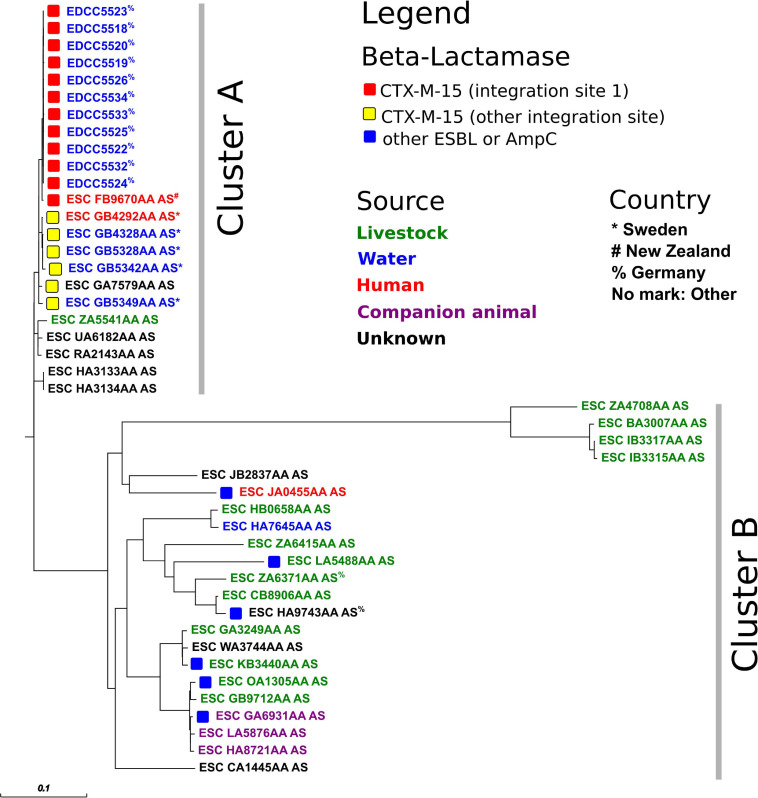
Core-genome-based phylogenetic tree of *E. coli* ST-949 isolates from this study and Enterobase (as of 8th June 2020). For a representation of the isolate sampling site, please see Supplementary Figure 1.

A whole-genome-based analysis of the *E. coli* ST-949 isolates from this study and those from Enterobase revealed two different findings (Figure 3 and Supplementary Table 1): Firstly, ST-949 isolates are divided into two different clusters. Cluster A (including our isolates) consists of isolates found in water, human, and livestock samples, while cluster B includes also isolates from companion animals. Secondly, only cluster A isolates contain CTX-M-15, while Cluster B isolates harbor either CTX-M alleles other than CTX-M-15, or AmpC beta-lactamases. Cluster B harbored two ST-949 isolates from Germany from livestock and an unknown source.

The *E. coli* ST-949 isolates from this study (Cluster A, *n* = 11) were highly related to ST-949 isolates found in New Zealand (ESC_FB9670AA_AS, human isolate) and Sweden (*n* = 4, water and human isolates, Figure 3). All these 16 isolates harbored a complex antibiotic resistance gene region including not only the *bla*_*CTX–M–15*_ gene, but also the fluoroquinolone resistance gene *qnrS1* and several different insertion sequences (Supplementary Figure 3). The antibiotic resistance region was inserted in the chromosome at an identical location in the isolates from this study and the isolates from New Zealand, while the isolates from Sweden harbored the identical region inserted at a different location of the chromosome. This finding indicates that the acquisition of *bla*_*CTX–M–15*_ in the two different clones was presumably from a different source and was independent in both clones.

The epidemiological link between Germany and New Zealand is not clear. It may indicate that the ST-949 clone found in Germany is present worldwide, but this is only an assumption as the total number of ST-949 isolates throughout the world is very low. It remains to be clarified whether ST-949 is an emerging ST and whether it is present in other sources.

The epidemiological link between ST-949 isolates from our study is partly explainable. All ST-949 river isolates originate from the same river (sampling sites 10, 11, 16, and 17), indicating a common source of contamination. Possible sources of contamination along the river might be either agriculture, two large university hospitals whose cleared wastewater end up in the river itself or human influence through tourism, as the river is frequently used for recreational purposes. Sampling site 6 is located close to the sampled river, indicating a possible contamination through the river by flooding. What is not completely clear, is the epidemiological link between sampling site 1 and the other sites. They are not interconnected by any water flows. A possible connection between those might have been movement of humans or animals (in particular birds).

ST-949 isolates have never been reported in Germany. Therefore, this is the first study detecting ST-949 *E. coli* in Germany. Its predominance in our study indicates that either ST-949 *E. coli* might resemble *E. coli* isolates only present in water sources or a new emerging multilocus ST in Germany. To prove this hypothesis, more studies are required.

## Conclusion

In this study, we characterized ESBL-producing *E. coli* isolates from water samples. Our results show that the main MLST type is ST-949, reported in only a few number of very recent publications. In addition, it has been associated with human disease. This indicates that it might be an emerging ST with human pathogenic potential that could spread through water sources.

## Data Availability Statement

The datasets presented in this study can be found in online repositories. The names of the repository/repositories and accession number(s) can be found below: https://www.ncbi.nlm.nih.gov/, PRJNA656216.

## Author Contributions

ED designed the study. AN and SH collected samples and data. ED performed antibiotic resistance determination. LF, AN, JF, and ED analyzed the data. LF, AN, and ED wrote the manuscript that was critically reviewed and approved by all authors.

## Conflict of Interest

The authors declare that the research was conducted in the absence of any commercial or financial relationships that could be construed as a potential conflict of interest.

## Footnotes

1 https://www.eucast.org/fileadmin/src/media/PDFs/EUCAST_files/Breakpoint_tables/v_11.0_Breakpoint_Tables.pdf

2 https://github.com/rrwick/Porechop

3 http://www.genomicepidemiology.org/

4 http://www.dwd.de and http://www.wetter.de
