# Supplementary material for: Clonal CTX-M-15-Producing Escherichia coli ST-949 Are Present in German Surface Water
Source: Front Microbiol. 2021 Apr 12;12:617349. doi: 10.3389/fmicb.2021.617349 (PMC8072356; doi:10.3389/fmicb.2021.617349)
Supplement: Supplementary file 1 [file Data_Sheet_1.docx]

Supplementary Material

To the manuscript entitled: Clonal CTX-M-15-producing *Escherichia coli* ST-949 are present in German Surface Water

# Supplementary Data

The supplementary material contains the following data:

- Supplementary Figure 1
- Supplementary Figure 2
- Supplementary Figure 3
- Supplementary Table 1
- Supplementary Table 2

# Supplementary Figures and Tables

## Supplementary Figures

**Supplementary Figure 1.** Geographic location of *E. coli* ST-949 isolates from Enterobase. For an interactive view of the figure, please see the following link: <https://microreact.org/project/vFTXgSa8cDV2hyPdf4wzvc>. The figure was produced using microreact (Argimón et al., 2016).

**
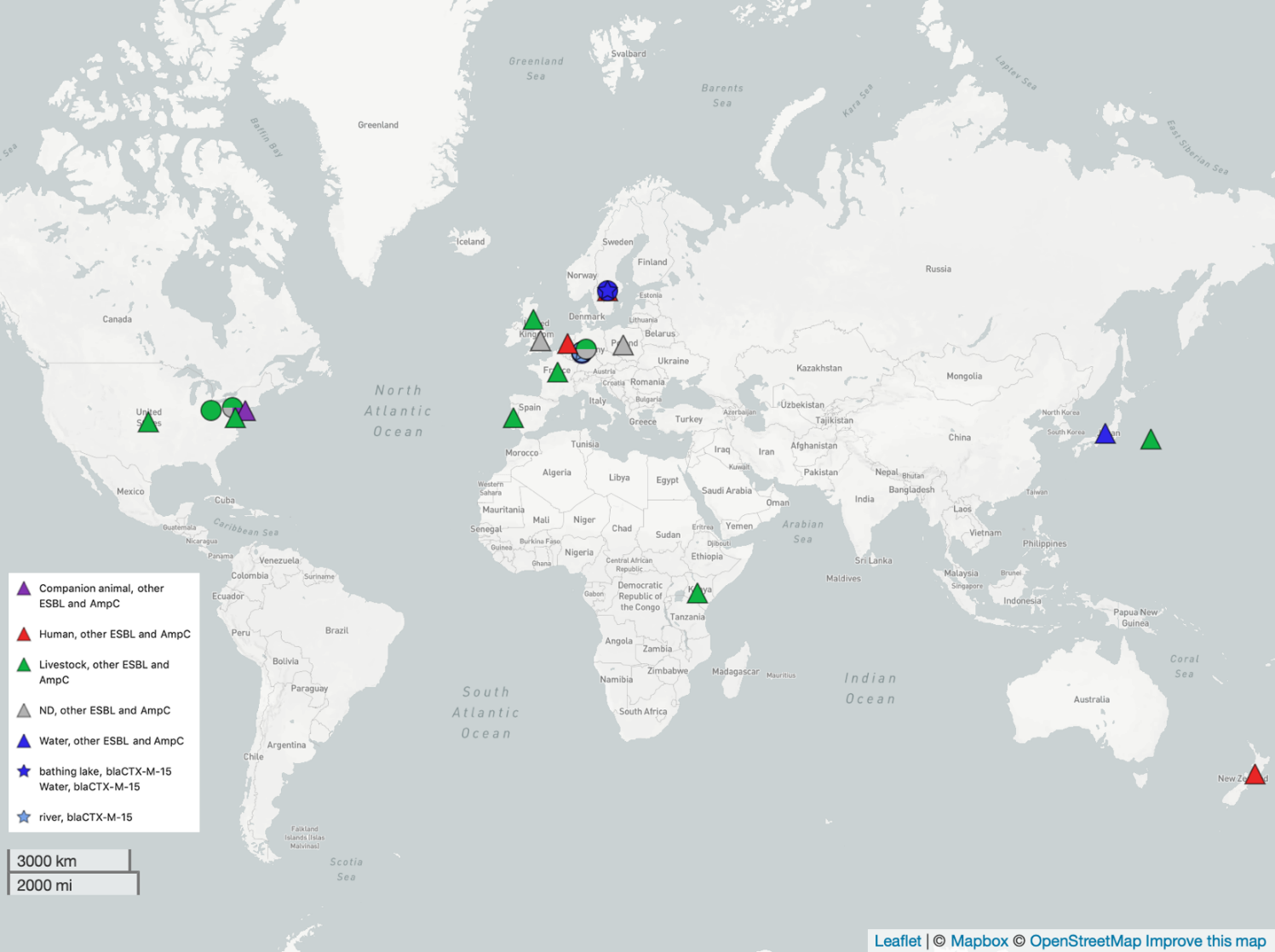
**

**Supplementary Figure 2.** Depiction of virulence determinants present in *E. coli* ST-949 isolates. Black, complete operon, grey, incomplete operon. Marked in blue are isolates from this study. Country of isolation: ^%^ Germany, # New Zealand, * Sweden, no mark: other countries.


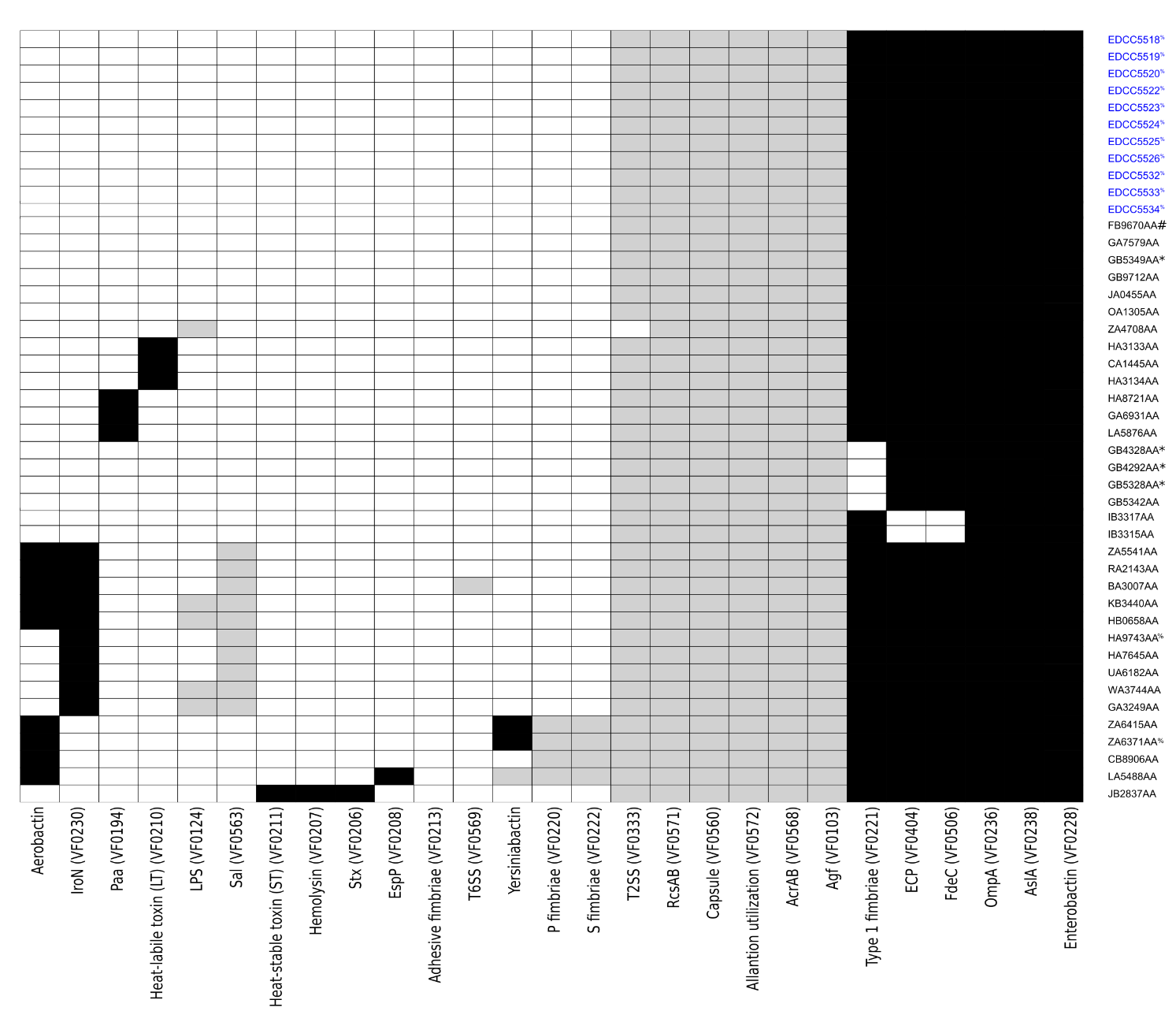


**Supplementary Figure 3.** Depiction of the antibiotic resistance cassette of ST-949 isolates from this study. IR = Inverted repeat.

**
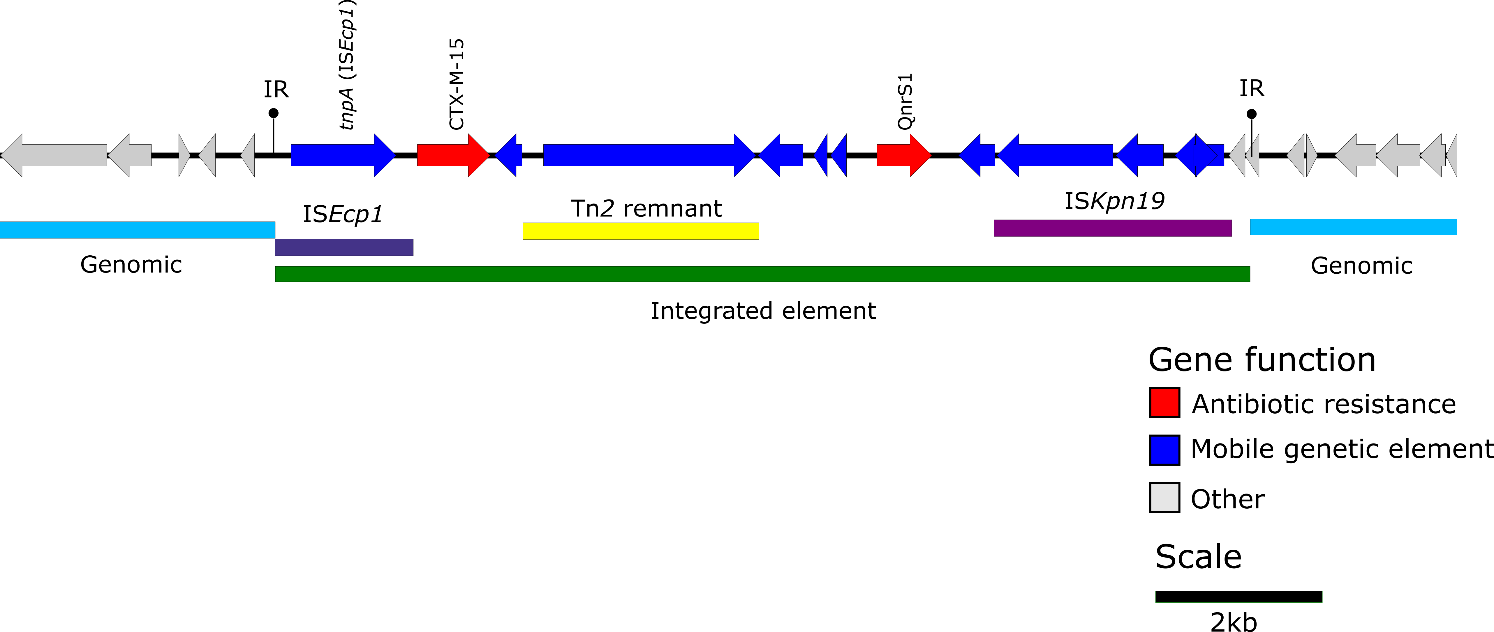
**

# Supplementary Tables

**Supplementary Table 1: Characteristics of ST-949 *E. coli* from Enterobase (as of 8^th^ June 2020)**

| Assembly barcode | Source | Collection  Year | Collection  Month | Country | Accession  number | ESBL/AmpC |
| --- | --- | --- | --- | --- | --- | --- |
| ESC_BA3007AA_AS | Livestock | 2013 | 7 | United States | SRS580037 |  |
| ESC_CA1445AA_AS | ND | ND | ND | ND | SRS5223556 |  |
| ESC_CB8906AA_AS | Livestock | 1977 | ND | United States | SRS5520993 |  |
| ESC_FB9670AA_AS | Human | 2016 | ND | New Zealand | ND | CTX-M-15 |
| ESC_GA3249AA_AS | Livestock | 2014 | ND | Australia | SRS1417001 |  |
| ESC_GA6931AA_AS | Companion animal | 2007 | 5 | United States | SRS5196868 | CMY-2 |
| ESC_GA7579AA_AS | ND | ND | ND | ND | SRS5196904 | CTX-M-15 |
| ESC_GB4292AA_AS | Human | 2013 | 7 | Sweden | SRS5196908 | CTX-M-15 |
| ESC_GB4328AA_AS | Water | 2013 | 10 | Sweden | SRS5196929 | CTX-M-15 |
| ESC_GB5328AA_AS | Water | 2013 | 10 | Sweden | SRS5696779 | CTX-M-15 |
| ESC_GB5342AA_AS | Water | 2013 | 10 | Sweden | DRS032761 | CTX-M-15 |
| ESC_GB5349AA_AS | Water | 2013 | 6 | Sweden | SRS1594037 | CTX-M-15 |
| ESC_GB9712AA_AS | Livestock | 2019 | 7 | United States | ERS1023680 |  |
| ESC_HA3133AA_AS | ND | ND | ND | ND | SRS5749144 |  |
| ESC_HA3134AA_AS | ND | ND | ND | ND | ERS1340918 |  |
| ESC_HA7645AA_AS | Water | ND | ND | Japan | SRS6238748 |  |
| ESC_HA8721AA_AS | Companion animal | 2012 | 4 | United States | SRS6420795 |  |
| ESC_HA9743AA_AS | ND | 2014 | ND | Germany | ND | CTX-M-1-like |
| ESC_HB0658AA_AS | Livestock | 2019 | 10 | United States | ND |  |
| ESC_IB3315AA_AS | Livestock | ND | ND | ND | SRS2028566 |  |
| ESC_IB3317AA_AS | Livestock | ND | ND | ND | SRS301924 |  |
| ESC_JA0455AA_AS | Human | ND | ND | Netherlands | ERS980259 | CTX-M-1 |
| ESC_JB2837AA_AS | ND | 2020 | 2 | United Kingdom | ERS644970 |  |
| ESC_KB3440AA_AS | Livestock | 2015 | ND | United Kingdom | ERS644969 | CMY-2 |
| ESC_LA5488AA_AS | Livestock | 2017 | ND | United States | SRS2042864 | CARB-2,CMY-2 |
| ESC_LA5876AA_AS | Companion animal | 2010 | 2 | United States | ERS1801949 |  |
| ESC_OA1305AA_AS | Livestock | 2015 | ND | Portugal | ERS1501247 | CTX-M-32 |
| ESC_RA2143AA_AS | ND | 2012 | 1 | Poland | ERS2055661 |  |
| ESC_UA6182AA_AS | ND | ND | ND | ND | SRS2535034 |  |
| ESC_WA3744AA_AS | ND | 2012 | 9 | United States | ERS3414862 |  |
| ESC_ZA4708AA_AS | Livestock | 2016 | 5 | Kenya | SRS4831424 |  |
| ESC_ZA5541AA_AS | Livestock | 2019 | ND | United States | SRS5196923 |  |
| ESC_ZA6371AA_AS | Livestock | 2004 | 3 | Germany | ERS3423052 |  |
| ESC_ZA6415AA_AS | Livestock | 2010 | 1 | France | ERS3423096 |  |

**Supplementary Table 2: Minimal inhibitory concentrations of the *E. coli* isolates. All isolates are multidrug-resistant (= resistant to ≥3 antibiotic classes).**

| Isolate | Sampling date | Ampicillin | Ampicillin/Sulbactam | Piperacillin/Tazobactam | Cefuroxim | Cefpodoxim | Cefotaxim | Ceftazidim | Imipenem | Meropenem | Gentamicin | Ciprofloxacin | Tigecycline | Fosfomycin | Nitrofurantoin | Trimethoprim/Sulfamethoxazol | ESBL confirmation test |
| --- | --- | --- | --- | --- | --- | --- | --- | --- | --- | --- | --- | --- | --- | --- | --- | --- | --- |
| EDCC  5518 | 19.06.  2018 | ≥32 | ≤2 | ≤4 | ≥64 | ≥8 | ≥64 | 4 | ≤0.25 | ≤0.25 | ≤1 | ≤0.25 | ≤0.5 | ≤16 | ≤16 | ≤20 | + |
| EDCC  5519 | 19.06.  2018 | ≥32 | ≤2 | ≤4 | ≥64 | ≥8 | ≥64 | 4 | ≤0.25 | ≤0.25 | ≤1 | ≤0.25 | ≤0.5 | ≤16 | ≤16 | ≤20 | + |
| EDCC  5520 | 19.06.  2018 | ≥32 | ≤2 | ≤4 | ≥64 | ≥8 | ≥64 | 4 | ≤0.25 | ≤0.25 | ≤1 | ≤0.25 | ≤0.5 | ≤16 | ≤16 | ≤20 | + |
| EDCC  5521 | 16.07.  2018 | ≥32 | 16 | ≤4 | ≥64 | ≥8 | ≥64 | ≤1 | ≤0.25 | ≤0.25 | ≤1 | ≤0.25 | ≤0.5 | ≤16 | ≤16 | ≤20 | + |
| EDCC  5522 | 17.07.  2018 | ≥32 | ≤2 | ≤4 | ≥64 | ≥8 | ≥64 | 4 | ≤0.25 | ≤0.25 | ≤1 | ≤0.25 | ≤0.5 | ≤16 | ≤16 | ≤20 | + |
| EDCC  5523 | 07.08.  2018 | ≥32 | ≤2 | ≤4 | ≥64 | ≥8 | ≥64 | 4 | ≤0.25 | ≤0.25 | ≤1 | ≤0.25 | ≤0.5 | ≤16 | ≤16 | ≤20 | + |
| EDCC  5524 | 07.08.  2018 | ≥32 | ≤2 | ≤4 | ≥64 | ≥8 | ≥64 | 4 | ≤0.25 | ≤0.25 | ≤1 | ≤0.25 | ≤0.5 | ≤16 | ≤16 | ≤20 | + |
| EDCC  5525 | 08.08.  2018 | ≥32 | ≤2 | ≤4 | ≥64 | ≥8 | ≥64 | 4 | ≤0.25 | ≤0.25 | ≤1 | ≤0.25 | ≤0.5 | ≤16 | ≤16 | ≤20 | + |
| EDCC  5526 | 08.08.  2018 | ≥32 | ≤2 | ≤4 | ≥64 | ≥8 | ≥64 | 4 | ≤0.25 | ≤0.25 | ≤1 | ≤0.25 | ≤0.5 | ≤16 | ≤16 | ≤20 | + |
| EDCC  5527 | 08.08.  2018 | ≥32 | ≤2 | ≤4 | ≥64 | ≥8 | 8 | ≤1 | ≤0.25 | ≤0.25 | ≤1 | 1 | ≤0.5 | ≤16 | ≤16 | ≥320 | + |
| EDCC  5528 | 08.08.  2018 | ≥32 | ≤2 | ≤4 | ≥64 | ≥8 | 8 | 4 | ≤0.25 | ≤0.25 | ≤1 | 0.5 | ≤0.5 | ≤16 | ≤16 | ≤20 | + |
| EDCC  5529 | 08.08.  2018 | ≥32 | 16 | ≤4 | ≥64 | ≥8 | ≥64 | 16 | ≤0.25 | ≤0.25 | ≤1 | 0.5 | ≤0.5 | ≤16 | ≤16 | ≤20 | + |
| EDCC  5530 | 08.08.  2018 | ≥32 | 16 | ≤4 | ≥64 | ≥8 | ≥64 | ≤1 | ≤0.25 | ≤0.25 | ≤1 | ≤0.25 | ≤0.5 | ≤16 | ≤16 | ≥320 | + |
| EDCC  5531 | 08.08.  2018 | ≥32 | ≥32 | 8 | ≥64 | ≥8 | ≥64 | 4 | ≤0.25 | ≤0.25 | ≤1 | 0.5 | ≤0.5 | ≤16 | ≤16 | ≤20 | + |
| EDCC  5532 | 08.08.  2018 | ≥32 | ≤2 | ≤4 | ≥64 | ≥8 | ≥64 | 4 | ≤0.25 | ≤0.25 | ≤1 | ≤0.25 | ≤0.5 | ≤16 | ≤16 | ≤20 | + |
| EDCC  5533 | 08.08.  2018 | ≥32 | ≤2 | ≤4 | ≥64 | ≥8 | ≥64 | 4 | ≤0.25 | ≤0.25 | ≤1 | ≤0.25 | ≤0.5 | ≤16 | ≤16 | ≤20 | + |
| EDCC  5534 | 08.08.  2018 | ≥32 | ≤2 | ≤4 | ≥64 | ≥8 | ≥64 | 4 | ≤0.25 | ≤0.25 | ≤1 | ≤0.25 | ≤0.5 | ≤16 | ≤16 | ≤20 | + |
| EDCC  5535 | 08.08.  2018 | ≥32 | ≤2 | ≤4 | ≥64 | ≥8 | ≥64 | 4 | ≤0.25 | ≤0.25 | ≤1 | ≥4 | ≤0.5 | ≤16 | ≤16 | ≤20 | + |
| EDCC  5536 | 08.08.  2018 | ≥32 | 16 | ≤4 | ≥64 | ≥8 | ≥64 | 4 | ≤0.25 | ≤0.25 | ≤1 | ≤0.25 | ≤0.5 | ≤16 | ≤16 | ≥320 | + |
| EDCC  5537 | 08.08.  2018 | ≥32 | 16 | ≤4 | ≥64 | ≥8 | ≥64 | 8 | ≤0.25 | ≤0.25 | ≤1 | ≥4 | ≤0.5 | ≤16 | ≤16 | ≥320 | + |
| EDCC  5538 | 08.08.  2018 | ≥32 | 16 | ≤4 | ≥64 | ≥8 | ≥64 | 8 | ≤0.25 | ≤0.25 | ≤1 | ≥4 | ≤0.5 | ≤16 | ≤16 | ≥320 | + |

**EDCC: Eugen Domann culture collection**

**Supplementary Table 3. Number of isolates resistant/susceptible to other antibiotics than beta-lactams**

|  | Imipenem | Meropenem | Gentamicin | Ciprofloxacin | Levofloxacin | Tigecycline | Fosfomycin | Nitrofurantoin | Trimethoprim/  Sulfamethoxazol |
| --- | --- | --- | --- | --- | --- | --- | --- | --- | --- |
| R | 0 | 0 | 0 | 4 | 4 | 0 | 0 | 0 | 5 |
| S | 21 | 21 | 21 | 17 | 17 | 21 | 21 | 21 | 16 |

Supplementary References

Argimón, S., Abudahab, K., Goater, R. J. E., Fedosejev, A., Bhai, J., Glasner, C., et al. (2016). Microreact: visualizing and sharing data for genomic epidemiology and phylogeography. *Microb. Genomics* 2, e000093. doi:10.1099/mgen.0.000093.
